# TIMP-1 is under regulation of the EGF signaling axis and promotes an aggressive phenotype in *KRAS*-mutated colorectal cancer cells: A potential novel approach to the treatment of metastatic colorectal cancer

**DOI:** 10.18632/oncotarget.11118

**Published:** 2016-08-08

**Authors:** Line S. Tarpgaard, Maj Sofie Ørum-Madsen, Ib J. Christensen, Cathrine Nordgaard, Julie Noer, Tormod K. Guren, Bengt Glimelius, Halfdan Sorbye, Tone Ikdahl, Elin H. Kure, Kjell M. Tveit, Hans J. Nielsen, Per Pfeiffer, Nils Brünner, José M. A. Moreira

**Affiliations:** ^1^ Department of Oncology, Odense University Hospital, Odense, Denmark and University of Southern Denmark, Odense, Denmark; ^2^ Faculty of Health and Medical Sciences, University of Copenhagen, Copenhagen, Denmark; ^3^ The Finsen Laboratory, Rigshospitalet, Copenhagen, Denmark and Biotech Research and Innovation Center (BRIC), University of Copenhagen, Copenhagen, Denmark; ^4^ Department of Oncology and K. G. Jebsen Centre for Colorectal Cancer Research, Oslo University Hospital Oslo, Norway; ^5^ Departments of Radiology, Oncology and Radiation Science, Uppsala University, Uppsala and Department of Oncology and Pathology, Karolinska Institutet, Stockholm, Sweden; ^6^ Department of Oncology, Haukeland University Hospital, Bergen, Norway; ^7^ Department of Clinical Science, University of Bergen, Bergen, Norway; ^8^ Department of Oncology, Oslo University Hospital, Oslo, Norway; ^9^ Department of Genetics, Oslo University Hospital, Oslo, Norway; ^10^ Department of Surgical Gastroenterology, Copenhagen University Hospital, Hvidovre, Denmark

**Keywords:** metastatic colorectal cancer, plasma TIMP-1, KRAS mutations, prognosis, prediction

## Abstract

It is now widely accepted that therapeutic antibodies targeting epidermal growth factor receptor (EGFR) can have efficacy in *KRAS* wild-type advanced colorectal cancer (CRC) patients. What remains to be ascertained is whether a subgroup of *KRAS*-mutated CRC patients might not also derive benefit from EGFR inhibitors. Metalloproteinase inhibitor 1 (TIMP-1) is a pleiotropic factor predictive of survival outcome of CRC patients. Levels of TIMP-1 were measured in pre-treatment plasma samples (*n* = 426) of metastatic CRC patients randomized to Nordic FLOX (5-fluorouracil and oxaliplatin) +/− cetuximab (NORDIC VII study). Multivariate analysis demonstrated a significant interaction between plasma TIMP-1 protein levels, *KRAS* status and treatment with patients bearing *KRAS* mutated tumors and high TIMP-1 plasma level (> 3rd quartile) showing a significantly longer overall survival if treated with cetuximab (HR, 0.48; 95% CI, 0.25 to 0.93). To gain mechanistic insights into this association we analyzed a set of five different CRC cell lines. We show here that EGFR signaling induces TIMP-1 expression in CRC cells, and that TIMP-1 promotes a more aggressive behavior, specifically in *KRAS* mutated cells. The two sets of data, clinical and *in vitro*, are complementary and support each other, lending strength to our contention that TIMP- 1 plasma levels can identify a subset of patients with *KRAS*-mutated metastatic CRC that will have benefit from EGFR-inhibition therapy.

## INTRODUCTION

Different types of treatment are available for patients with advanced metastatic colorectal cancer (mCRC), including targeted biological treatment. Nonetheless, there is a continued unmet need for effective, and fast, point-of-care tests for predictive biomarkers in order to select the right treatment for individual patients. Epidermal growth factor receptor (EGFR) is recognized as a key factor in colorectal cancer (CRC) development and progression due to its effects on tumor-promoting processes, such as cellular proliferation, survival, and motility [1–5]. It is therefore not surprising that this receptor tyrosine kinase has become a major therapeutic target, with several approved anti-EGFR drugs currently being used in the clinic, including the antibodies cetuximab and panitumumab [6, 7]. However, only few patients have long-term responses to these agents, with clinical benefit almost always curtailed by the development of acquired drug resistance. At the moment, it is thought that anti-EGFR antibodies function at two levels: i) by preventing ligand-induced activation of downstream effectors that mediate intracellular signaling pathways, such as the RAS/RAF mitogen-activated protein kinase (MAPK) pathway, or phosphoinositide 3-kinase (PI3K)/ protein kinase B (AKT) pathway [4, 6], and ii) by triggering antibody-dependent cellular cytotoxicity (ADCC) [8, 9]. As one may expect, resistance to anti-EGFR antibodies can occur through functional alterations at any of these levels. Thus, activating mutations in downstream effectors, such as *RAS*, *RAF*, or *PI3KCA,* can result in persistent growth signaling, and provide a functional bypass of EGFR-blockade. In addition, *KRAS* mutations can also impair the therapeutic effect of cetuximab-induced ADCC [9, 10]. Concordantly, only *KRAS* wild-type (wt) patients seem to derive full benefit from anti-EGFR therapy, and as a consequence, clinical use of cetuximab and panitumumab in CRC is currently restricted to patients bearing *KRAS* wt tumors [11–16]. However, there are a number of discordant findings, with *in vitro* and *in vivo* data suggesting that some patients with *KRAS* mutated tumors may actually still have benefit from cetuximab treatment [12, 13, 17–19]. Conversely, response rates to cetuximab combination regimens are about 40% in the best of cases – including patients with no mutations at all in *KRAS*, *BRAF*, *NRAS*, and *PIK3CA* exon 20 [12]. Clearly, there are a number of confounding factors, such as the exact nature of *KRAS* mutations (G13D or other), levels of EGFR expression, EGFR mutations, first or later lines of therapy, chemotherapeutic backbone (irinotecan or oxaliplatin), or even administration regimen (infusion or bolus), which all seem to interplay and ultimately can affect the outcome of anti-EGFR therapy in CRC patients. Thus, additional predictive biomarkers are needed to improve stratification of patients with mCRC to EGFR inhibitor therapies.

TIMP-1 is a 28 kDa glycoprotein that can be found in the extracellular compartment in several tissues, and is present in various body fluids [20]. TIMP-1 is one of four (TIMP-1 through 4) human natural endogenous inhibitors of matrix metalloproteinases (MMPs), a group of peptidases involved in degradation of the extracellular matrix. In addition to its function as inhibitor of MMPs, TIMP-1 can have tumor-promoting effects, including stimulation of cell proliferation, induction of anti-apoptotic signaling, and support of angiogenesis [21–24]. Plasma TIMP-1 is elevated in patients with CRC [20, 25] and high plasma TIMP-1 levels have consistently been associated with poor prognosis in patients with primary or advanced CRC [25–29]. In this respect it should be noted that an association between TIMP-1 expression and EGFR signaling has also been observed in various cellular contexts [30–35], and is found to occur, at least under certain circumstances, via NF-κB signaling, in a MEK-independent manner [36]. Taken together, these data raised the possibility of an association between EGFR signaling, TIMP-1 expression and response to anti-EGFR agents. We show here that TIMP-1 plasma levels were associated with patient outcome in mCRC, and that patients bearing *KRAS*-mutated tumors and high TIMP-1 plasma level (> 3rd quartile) showed a significantly longer overall survival when treated with cetuximab (HR 0.48; 95% CI, 0.25 to 0.93), as compared to patients with *KRAS* mutated tumors not treated with cetuximab. These results were substantiated in preclinical cellular models, where we found that exposure of CRC cell lines to recombinant TIMP-1 (rTIMP-1) promoted a more aggressive behavior, specifically in *KRAS* mutated cells. Taken together, these data indicate that plasma TIMP-1 levels, which can be measured with a simple and non-invasive point-of-care test, may be useful for selection of patients bearing *KRAS* mutated tumors that will derive benefit from EGFR-inhibition therapy.

## RESULTS

Given that previous work from our group, as well as from other groups, had shown that TIMP-1 can be predictive of outcome in CRC [20, 29–31], and that TIMP-1 can promote cancer cell survival through the PI3K/AKT signaling axis [32, 33], we reasoned that TIMP-1 could influence response to anti-EGFR therapy. To address this question, we tested interactions between plasma TIMP-1 levels and EGFR targeted treatment in a clinical setting.

### Pre-treatment plasma TIMP-1 and associations to progression-free survival and overall survival

Plasma samples from patients enrolled in the NORDIC VII study were available to us. The NORDIC VII study was a three-arm, phase III prospective randomized clinical trial, of anti-EGFR therapy (cetuximab) [34]. The design of this study, A versus A+B where cetuximab is B, lends itself to studies on predictive biomarkers for cetuximab effects.

**Figure 1:**
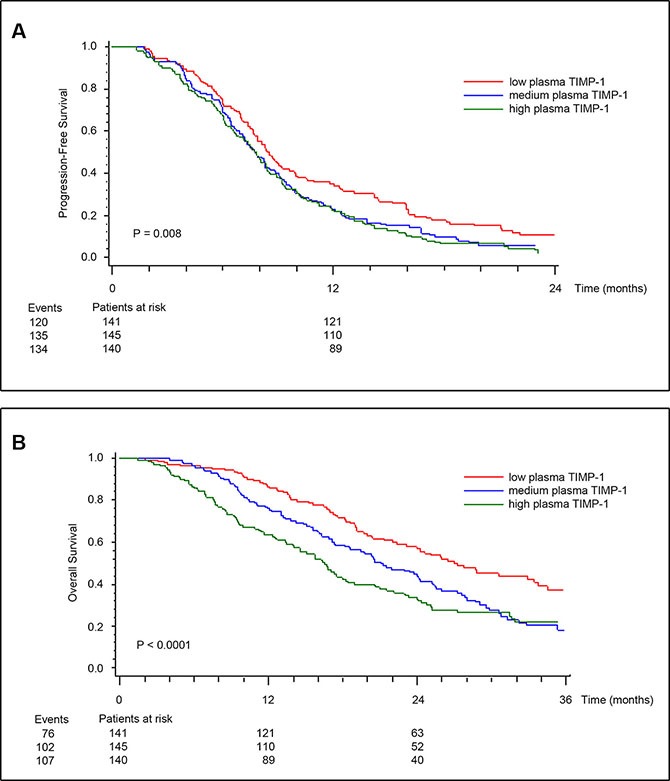
Kaplan-Meier estimates of survival probabilities for PFS (A), and OS (B) stratified by pre-treatment plasma TIMP-1 Plasma TIMP-1 was categorized according to its tertile levels. *P* values calculated by log-rank test.

To address the possibility of an association between EGFR signaling, TIMP-1 expression and response to anti-EGFR agents, we measured total plasma TIMP-1 levels (free and in complex with matrix metalloproteinase) in the 426 samples that were available from the study (Figure S1), using an ELISA assay developed in-house [35]. The two study populations (+/− cetuximab) were similar and not different from the total intention to treat population of 566 patients. The median pre-treatment plasma TIMP-1 was 269 ng/mL (58 to 1318 ng/mL) with no differences between the two treatment groups (*P* = 0.97). Tumors were *KRAS* mutated in 147 patients (39%). There was no association between pre-treatment plasma TIMP-1 and gender, or number of metastatic sites. There were statistically significant associations between pre-treatment plasma TIMP-1 and WHO performance status (PS), location of the primary tumor, previous adjuvant chemotherapy, *KRAS* and *BRAF* status. The highest plasma TIMP-1 values were found in patient with high WHO PS, primary tumor in colon, no adjuvant therapy, *KRAS* wild-type tumors and *BRAF* mutated tumors (Table 1). Univariate Cox analyses including all patients showed that high pre-treatment plasma TIMP-1 was significantly associated with shorter progression-free survival (PFS) (HR, 1.22; 95% CI, 1.07 to 1.39; *P* = 0.003), and overall survival (OS) (HR, 1.55; 95% CI, 1.33 to 1.80; *P* < 0.0001) (Table 2). Kaplan-Meier estimates of survival probabilities for PFS and OS stratified by pre-treatment plasma TIMP-1 levels are shown in Figure 1A and 1B, respectively. Multivariate analysis (plasma TIMP-1, *KRAS* and *BRAF* status, serum CRP, serum CEA, WHO PS, number of metastatic sites, age, and gender) demonstrated that high plasma TIMP-1 was an independent biomarker of short OS (HR, 1.35; 95% CI, 1.12 to 1.62; *P* = 0.016) while a significant interaction between plasma TIMP-1 and *KRAS* mutational status could not be demonstrated (*P* = 0.47). Plasma TIMP-1 was not significantly associated to PFS in this multivariate model (*P* = 0.82).

**Table 1:** Demographic and baseline clinical characteristics of the 426 patients with metastatic colorectal cancer included in the Nordic VII study for whom pre-treatment plasma TIMP-1 was measured

Variable		*N* (%)	Median plasma TIMP-1 (range)	*P*-value for Wilcoxon test
Gender	Males	253 (59)	278 (58–1318)	0.14
	Females	173 (41)	259 (86–1317)	
WHO PS	0	295 (69)	237 (58–1317)	< 0.0001
	1	118 (28)	339 (123–1317)	
	2	13 (3)	524 (228–1318)	
Location	Colon	250 (59)	282 (58–1317)	0.018
	Rectum	176 (41)	251 (84–1318)	
Number of metastatic sites	1	124 (29)	232 (87–1318)	0.046
	> 1	302 (71)	277 (58–1317)	
Adjuvant chemotherapy	Yes	38 (9)	217 (87–461)	< 0.0001
	No	388 (91)	280 (58–1318)	
*KRAS*	WT	228 (54)	280 (84–1318)	0.012^*^
	Mutant	149 (35)	237 (87–1027)	
	Missing	49 (12)	395 (58–11317)	
*BRAF*	WT	304 (71)	257 (84–1318)	0.35^*^
	Mutant	38 (9)	285 (105–1314)	
	Missing	84 (20)	339 (58–1317)	

* *P*-value for complete data.

**Table 2:** Univariate and multivariate cox analyses of PFS and OS in the 426 patients with metastatic colorectal cancer (389 with progression, 285 deaths) included in the NORDIC VII study according to pre-treatment plasma TIMP-1 and clinical parameters

	Progression-free Survival						Overall Survival					
	Univariate Cox analyses			Multivariate Cox analyses			Univariate Cox analyses			Multivariate Cox analyses		
	HR	95% CI	*p*-value	HR	95% CI	*p*-value	HR	95% CI	*p*-value	HR	95% CI	*p*-value
Cetuximab Yes vs No	1.05	0.85–1.29	0.68	0.50^1^	0.27–0.91	^b^	1.09	0.85–1.40	0.48	0.48^1^	0.25–0.93	^a^
				1.14^2^	0.80–1.63					1.08^2^	0.72–1.63	
				1.33^3^	0.81–2.19					2.16^3^	1.07–4.33	
				1.27^4^	0.87–1.84					0.83^4^	0.52–1.03	
Plasma TIMP-1 (log)^*^	1.22	1.07–1.39	0.003	0.84^5^	0.63–1.12	^b^	1.55	1.33–1.80	< 0.0001	1.04^5^	0.76–1.42	^a^
				1.04^6^	0.80–1.34					1.59^6^	1.17–2.15	
				2.15^7^	1.04–4.42					4.45^7^	1.73–11.48	
				1.15^8^	0.81–1.62					1.22^8^	0.83–1.79	
Age per 10 years	0.94	0.84–1.04	0.23	0.92	0.82–1.03	0.14	0.97	0.86–1.09	0.60	1.00	0.88–1.14	0.97
Gender, Female vs. Male	1.08	0.88–1.32	0.46	0.92	0.74–1.13	0.41	0.94	0.74–1.19	0.61	1.16	0.90–1.49	0.26
*BRAF* Mutant vs. WT	2.00	1.41–2.82	< 0.0001	1.73	1.29–2.33	< 0.0001	3.31	2.30–4.77	< 0.0001	4.74	3.10–7.23	< 0.0001
*KRAS* Mutant vs WT	1.12	0.90–1.39	0.30	1.04^9^	0.74–1.46	^b^	0.98	0.76–1.27	0.88	1.14^9^	0.78–1.66	^a^
				2.37^10^	1.29–4.37					2.56^10^	1.30–5.06	
				1.34^11^	0.97–1.85					1.76^11^	1.16–2.66	
				1.27^12^	0.74–2.20					0.67^12^	0.33–1.41	
Metastatic sites > 1 vs 1	1.41	1.13–1.76	0.0026	1.43	1.13–1.81	0.0034	1.59	1.22–2.08	0.0006	1.60	1.20–2.14	0.0015
WHO PS ≥ 1 vs 0	1.61	1.30–1.99	< 0.0001	1.33	1.05–1.68	0.020	1.88	1.47–2.39	< 0.0001	1.52	1.16–1.98	0.0023
Serum CRP, Elevated vs. normal	1.50	1.22–1.85	0.0002	1.44	1.12–1.86	0.0046	1.61	1.25–2.05	0.0002	1.27	0.94–1.71	0.11
Serum CEA, Elevated vs. normal	1.63	1.24–2.14	0.0004	1.39	1.03–1.88	0.034	1.93	1.38–2.71	0.0001	1.70	1.19–2.44	0.0038

HR = Hazard ratio. CI = Confdence interval.

* Plasma TIMP-1 was included as a log transformed continuous variable (log base 2).

a *P* = 0.004 for the interaction Treatment X *KRAS* X TIMP-1.

b *P* = 0.096 for the interaction Treatment X *KRAS* X TIMP-1.

1 HR for Cetuximab vs no cetuximab for *KRAS* mutant and TIMP-1 level at the 3rd quartile (409 ng/ml).

2 HR for Cetuximab vs no cetuximab for *KRAS* WT and TIMP-1 level at the 3rd quartile (409 ng/ml).

3 HR for Cetuximab vs no cetuximab for *KRAS* mutant and TIMP-1 level at the 1st quartile (201 ng/ml).

4 HR for Cetuximab vs no cetuximab for *KRAS* WT and TIMP-1 level at the 1st quartile (201 ng/ml).

5 HR for 2-fold difference TIMP-1 levels for mutant *KRAS* treated with Cetuximab.

6 HR for 2-fold difference TIMP-1 levels for WT *KRAS* treated with Cetuximab.

7 HR for 2-fold difference TIMP-1 levels for mutant *KRAS* not treated with Cetuximab.

8 HR for 2-fold difference TIMP-1 levels for WT not treated with Cetuximab.

9 HR for *KRAS* mutant vs WT receiving cetuximab and TIMP-1 level at the 3rd quartile (409 ng/ml).

10 HR for *KRAS* mutant vs WT not receiving cetuximab and TIMP-1 level at the 3rd quartile (409 ng/ml).

11 HR for *KRAS* mutant vs WT receiving cetuximab and TIMP-1 level at the 1st quartile (201 ng/ml).

12 HR for *KRAS* mutant vs WT not receiving cetuximab and TIMP-1 level at the 1st quartile (201 ng/ml).

For OS, a signifcant interaction between *KRAS*, TIMP-1 level and treatment with Cetuximab is demonstrated, *P* = 0.006 in a multivariable model, for PFS the same analysis gives *p* = 0.078, although not signifcant, the analysis is retained.

### Pre-treatment plasma TIMP-1 and benefit from cetuximab treatment

**Figure 2:**
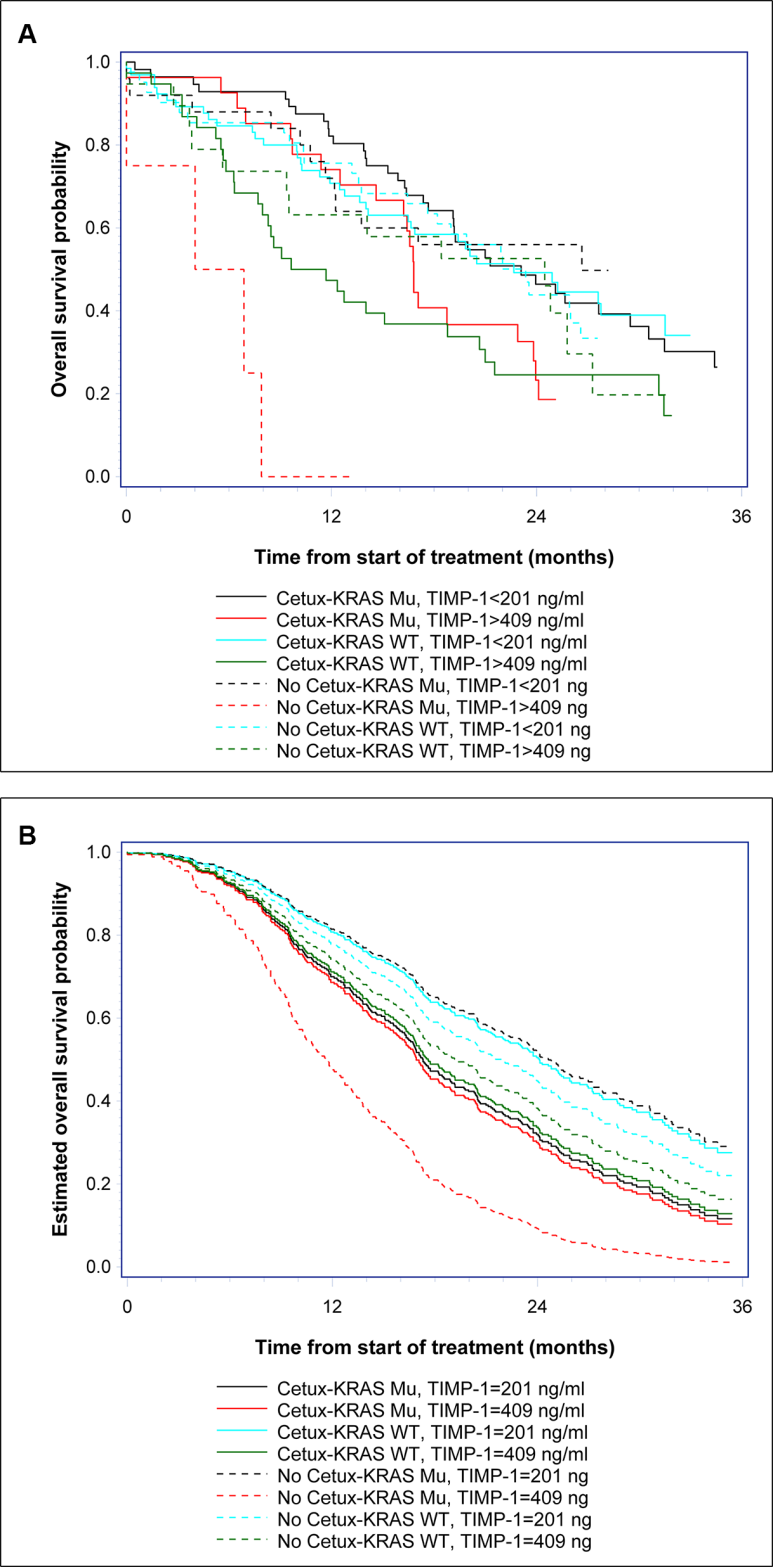
Kaplan-Meier estimates of survival probabilities (**A**) OS probabilities were estimated for patients treated, or not, with cetuximab (+/− cetuximab) stratified by *KRAS* status (*KRAS* wt or mutant) and TIMP-1 level, below 201 ng/ml (first quartile) or above 409 ng/ml (third quartile). (**B**) The estimated survival probabilities based on the multivariable Cox regression model. The covariates are set to: CEA status (elevated), CRP (elevated), gender (male), age (70 years), multiple metastatic sites, good performance status (WHO PS 0–1), *BRAF* wt, treatment (cetuximab or not), *KRAS* status (wt or mutant), and TIMP-1 level equal to 201 ng/ml (first quartile) or equal to 409 ng/ml (third quartile).

To determine if there was an association between TIMP-1 and response to cetuximab, we examined TIMP-1 plasma levels in patients responding, or not, to cetuximab. Pre-treatment plasma TIMP-1 levels were not significantly different in non-responders versus responders (median 282 μg/l vs. 260 μg/l; OR, 1.16; 95% CI, 0.91 to 1.49; *P* = 0.23) when looking at all patients and independently of *KRAS* status. Multivariate analysis showed a trend for an association between RR and pre-treatment plasma TIMP-1 (OR, 1.34; 95% CI, 0.99 to 1.82; *P* = 0.059). A significant interaction between RR, cetuximab treatment, *KRAS* mutational status, and TIMP-1 could not be shown (*P* = 0.48). The results of the multivariate model for OS including pre-treatment plasma TIMP-1, *KRAS* and *BRAF* status, age, gender, CRP, CEA, WHO PS, number of metastatic sites and treatment, or not, with cetuximab are shown in Table 2. A significant 3-ways interaction between treatment (+/− cetuximab), *KRAS* mutational status, and plasma TIMP-1 baseline level was demonstrated (*P* = 0.006). The HR for plasma TIMP-1 for patients with *KRAS* mutant tumors not treated with cetuximab was 4.45 (95% CI, 1.73 to 11.48) compared to 1.04 (95% CI, 0.76 to 1.42) if treated with cetuximab. A comparison of patients treated with cetuximab versus those not treated with cetuximab for the *KRAS* mutant subgroup showed a longer OS (HR, 0.48, 95% CI, 0.25 to 0.93) if the plasma TIMP-1 level was relatively high (3rd quartile), whereas the opposite was found for those with low levels of plasma TIMP-1. There was no significant interaction between plasma TIMP-1 levels, treatment, and OS in patients with *KRAS* wild-type tumors (Table 2). Multivariate analysis of PFS could not demonstrate a similar significant association to plasma TIMP-1 (*P* = 0.078), however the analysis suggests a pattern similar to that seen for OS (Table 2). The above described effects between plasma TIMP-1 levels, treatment and OS, are illustrated in Figure 2A and 2B, respectively. Patients were stratified into eight groups according to treatment (received cetuximab or not), *KRAS* status (wt or mutant), and TIMP-1 level (below 201 ng/ml or above 409 ng/ml). Kaplan-Meier plots of OS showed that *KRAS* mutated patients, with plasma TIMP-1 levels above 409 ng/ml had a significantly worse outcome if not treated with cetuximab (stipled red line) as compared to those that did receive cetuximab (solid red line) (Figure 2A). Conversely, *KRAS* mutated patients, with plasma TIMP-1 levels below 201 ng/ml showed no significant difference in outcome, whether treated or not with cetuximab (solid and stipled black lines, respectively) (Figure 2A). Estimation of OS probabilities based on the full multivariable Cox regression model (taking into account the covariates CEA and CRP status, gender, age, multiple metastatic sites, performance status, and *BRAF* status), verified the association between *KRAS* status, plasma TIMP-1 levels, and response to cetuximab (Figure 2B, compare *KRAS* mutated patients, with high plasma TIMP-1 levels, stipled red line, with those that received cetuximab, solid red line).

### EGF induces TIMP-1 expression in CRC cell lines

**Figure 3:**
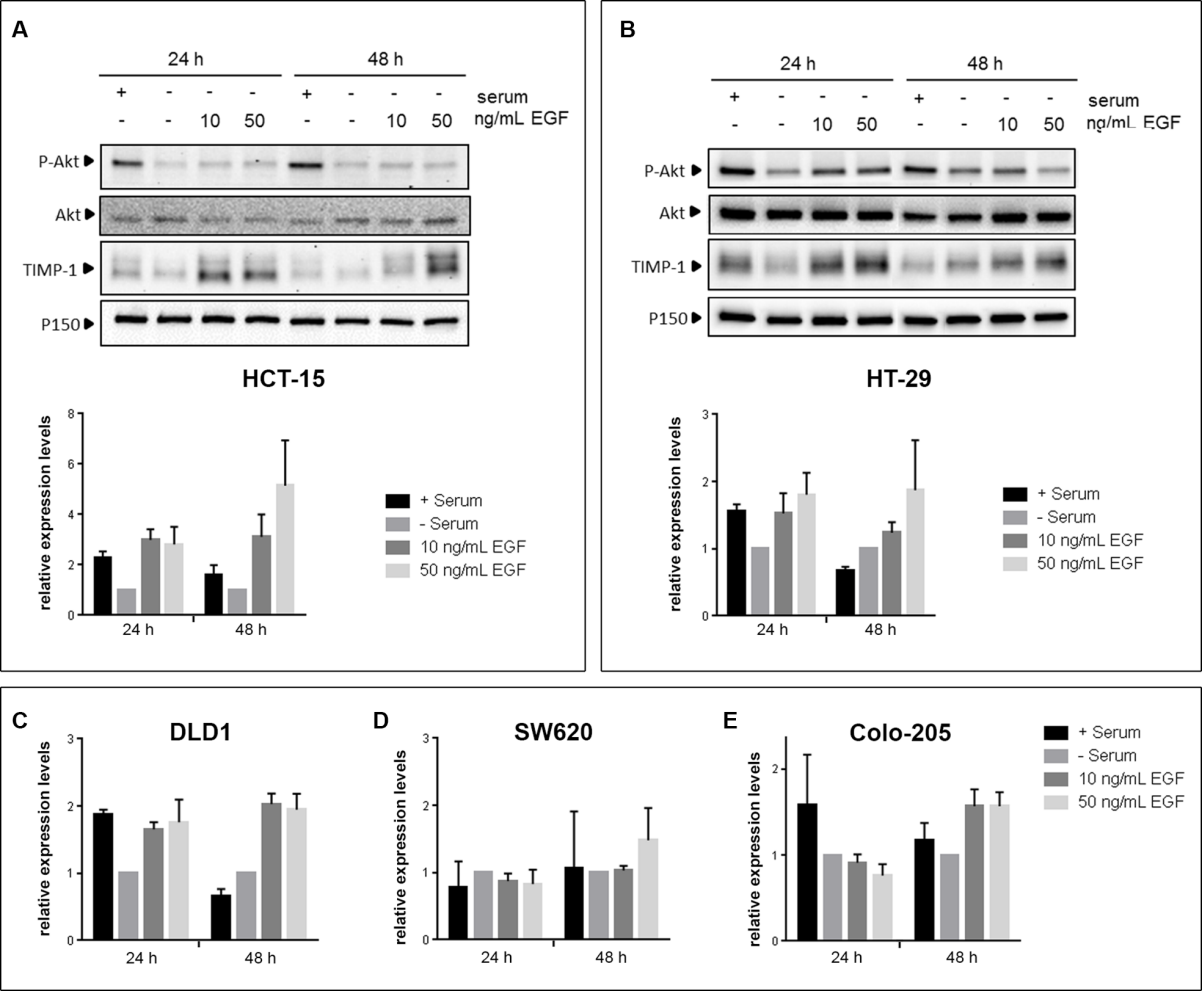
EGF induces TIMP-1 expression in CRC cells CRC cell lines were serum-deprived for 24 h prior to being stimulated with either 10 ng/mL or 50 ng/mL EGF for 24h and 48h. Controls, cultured with or without serum, were included in parallel. Immunoblotting of cell lysates was carried out using antibodies against P-AKT (Ser374), total AKT, TIMP-1 and p150^Glued^ (normalizing control). (**A** and **B**) Upper panels: immunoblots of HT-29 and HCT-15, respectively, lower panel: graph depicting pooled densitometry measurements of TIMP-1 levels relative to those of p150^Glued^. Data points are presented as mean ± SEM of triplicate experiments. (**C**–**E**) graph depicting pooled densitometry measurements of TIMP-1 levels relative to those of p150^Glued^ in immunoblots from DLD-1, SW620, and Colo-205, respectively.

To gain mechanistic insights into the association we found between TIMP-1 expression and benefit from cetuximab treatment in KRAS-mutated patients, we analyzed the effect(s) of TIMP-1 on CRC cells. Previous studies have shown that EGF stimulates TIMP-1 expression in extravillous trophoblasts and thyroid carcinoma cells [36, 37]. To ascertain the relevance of this interaction in CRC, we investigated the effect of EGF stimulation on TIMP-1 expression in cellular models of CRC. We analyzed five different CRC cell lines: SW620 (*KRAS* G12V, *BRAF* wt), Colo-205 (*KRAS* wt, *BRAF* V600E), HT-29 (*KRAS* wt, *BRAF* V600E), HCT-15 (*KRAS* G13D, *BRAF* wt), and DLD-1 (*KRAS* G13D, *BRAF* wt), for TIMP-1 expression upon stimulation with EGF. As shown in Figure 3, four of the five cell lines (Colo-205, HT-29, HCT-15, and DLD-1, respectively) showed increased, albeit to a varying degree, TIMP-1 expression upon stimulation with EGF. This effect was dose-dependent, as stimulation with 50 ng/mL EGF generally elicited relatively higher levels of TIMP-1 expression than 10 ng/mL (Figure 3, compare 10 ng/mL EGF with 50 ng/mL EGF), and was not associated with *KRAS* or *BRAF* status. In all cases phosphorylation of AKT at Ser374 was used as a measure of functional EGF signaling. In the cases of HCT-15 and HT-29 we found high basal levels of AKT phosphorylation even under serum-starvation growth conditions, consistent with the presence of activating mutations in *BRAF* and *KRAS*, respectively (Figure 3A and 3B, respectively). However, we could observe increased AKT phosphorylation after 24 h of ligand stimulation of EGFR, indicating that the stimulatory potential of the signaling pathway was maintained, and thus the increase in TIMP-1 expression following EGF stimulation could be ascribed to stimuli transduced through the EGFR signaling axis in HCT-15 and HT-29. Following 48 h of EGF stimulation, we observed a continued dose-dependent EGF-induced increase in TIMP-1 expression (Figure 3A–3C). Given the variable range in relative increases in TIMP-1 expression between the various cell lines in response to EGF stimulation, we analyzed EGFR expression in the five CRC cell lines we used. We found that these cells lines had very different levels of EGFR expression (Figure S2), and that the levels of EGFR expression in the five cell lines were consistent with the effect of EGF on TIMP-1 expression we had observed. Thus, HT29 (strong EGFR expression), and HCT15 and DLD1 (moderate EGFR expression) cells, displayed up-regulation of TIMP-1 expression upon exposure to EGF, whereas Colo-205 (weak EGFR expression), and SW620 (no detectable EGFR), showed very limited or no increase in TIMP-1 expression, respectively (Figure 3). We concluded that, overall, TIMP-1 expression is under regulation of the EGF-EGFR signaling axis in the cellular models of CRC we analyzed.

### TIMP-1 promotes colony formation in soft agar in *KRAS*-mutated cells only

**Figure 4:**
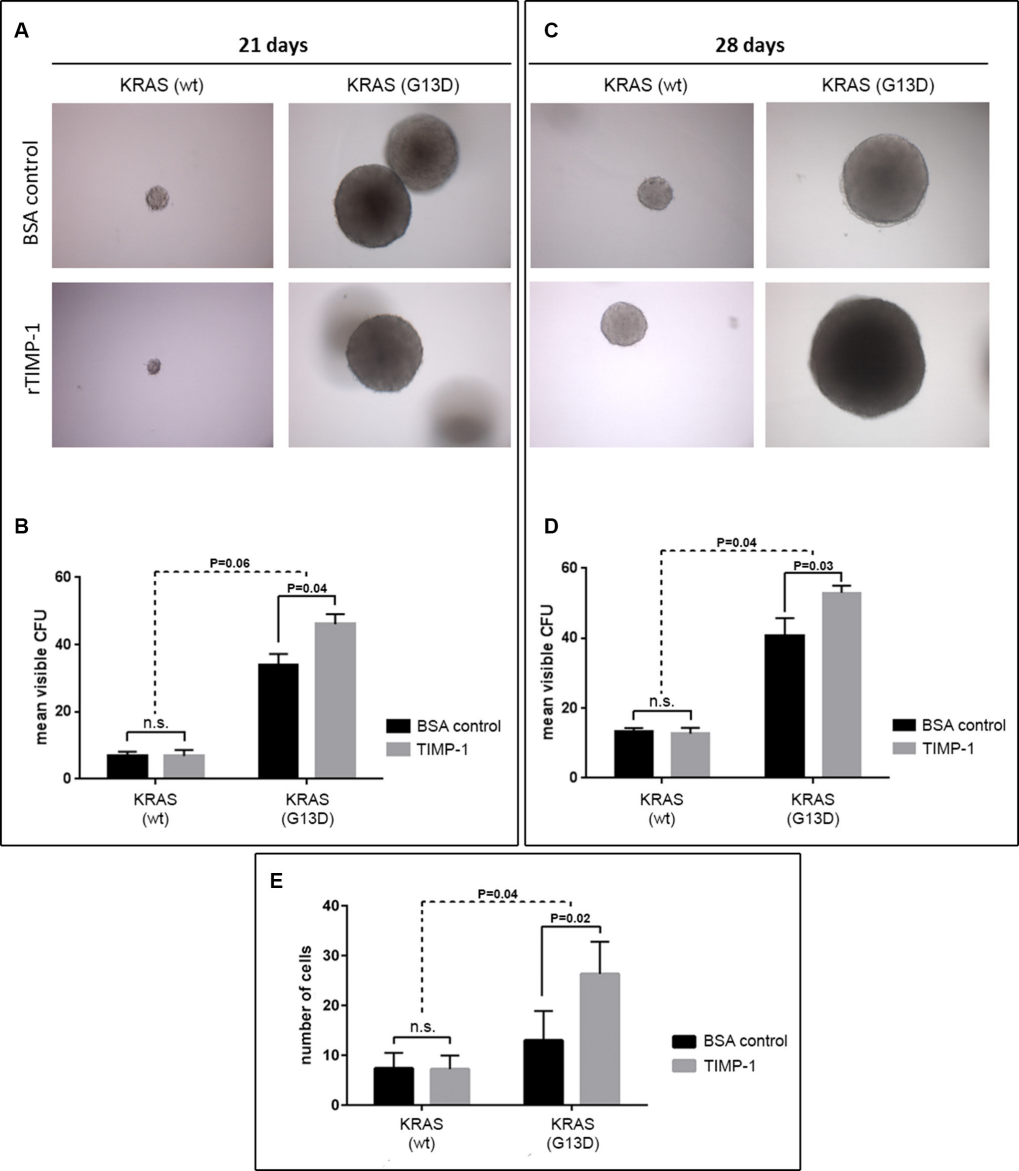
TIMP-1 promotes malignant behavior of *KRAS* mutated cells (**A**–**D**) TIMP-1 promotes colony formation in soft agar in *KRAS*-mutated cells. Tumorsphere formation was quantified [in colony forming units (CFUs)] for DLD-1 clones bearing either a *KRAS*-mutated allele (KRAS G13D) or a wild-type allele (KRAS wt) after 21 and 28 days of growth, in the presence of either rTIMP-1 (5 mg/mL) or BSA (control). Visible colonies were counted by two independent observers. Data represents mean ± SEM (error bars), of triplicate experiments. Significance was evaluated by two-way ANOVA with Sidak's multiple comparison post-test. Images were captured at 10 × magnification. (**E**) DLD-1 isogenic cell lines were serum-starved for 24 h before assessing the effect of TIMP-1 (5 mg/mL) on invasion in a 14-hour Boyden chamber invasion assay. Graph depicts the mean number of invaded cells of triplicate experiments. Invaded cells were counted independently by two different observers. Significance was determined by two-way ANOVA with Sidak's multiple comparison post-test. Images were acquired at 10× magnification.

In order to further assess the biological effects of TIMP-1 on CRC cells, and its association to *KRAS* mutational status, we performed an anchorage-independent growth analysis of a matched pair of isogenic DLD-1 cell clones (KRAS G13D and *KRAS* wt), in which either the wild-type or mutant *KRAS* allele has been disrupted [38]. We did a comparative soft-agar colony formation assay where we found that in the DLD-1 cell clone with the wild-type *KRAS* allele (KRAS wt), the number of colony forming units (CFU) formed after 21 or 28 days incubation, respectively, was not affected by the continuous presence of TIMP-1 in the growth media (5 μg/mL rTIMP-1) (Figure 4A and 4C, 21 days *P* > 0.999; 28 days *P* = 0.98, respectively). However, in the case of the DLD-1 clone with the *KRAS*-mutated allele (KRAS G13D), we observed an increase in the number of formed cell foci after 21 and 28 days incubation, respectively (Figure 4A and 4C, 21 days *P* = 0.04; 28 days *P* = 0.03, respectively). As expected, cells bearing the *KRAS* G13D mutated allele displayed increased ability to form colonies in soft agar, when compared to the clone with the *KRAS* wt allele (Figure 4B and 4D, respectively). The interaction between cell line and TIMP-1 exposure was non-significant at 21 days (*P* = 0.06), but it was significant after 28 days (*P* = 0.04).

### TIMP-1 enhances CRC cell invasion in a *KRAS* mutation dependent manner

A previously published study reported that TIMP-1 can induce a more aggressive phenotype in pancreatic ductal cells, but does so specifically in *KRAS*-mutated (G12D) cells [39]. To determine if TIMP-1 could promote invasion in a *KRAS*-dependent manner in CRC cells, we stimulated the matched pair of isogenic DLD-1 cell clones (KRAS G13D and KRAS wt) with exogenously added recombinant TIMP-1 (rTIMP-1; 5 μg/mL) [40] and compared the invasive potential of these cells to that of a control group (no added rTIMP-1), in a Boyden chamber invasion assay. We found an interaction between addition of rTIMP-1 and *KRAS* mutational status (Figure 4E). Whereas the DLD-1 cell clone bearing a wt-type *KRAS* allele showed no significant difference in invasive potential in the presence, or not, of TIMP-1 (Figure 4E; *P* = 0.999), *KRAS* mutated cells (KRAS G13D) responded to the presence of TIMP-1 in the growth medium (5.0 μg/mL rTIMP-1), becoming significantly more invasive (Figure 4E; KRAS G13D *P* = 0.0152). The invasive potential of DLD-1 *KRAS* mutated cells upon stimulation with TIMP-1, compared with the BSA control, was significantly different to that of *KRAS* wt cells (*P* = 0.0328) (Figure 4E). These results indicate that under the conditions of the assay, TIMP-1 promotes invasion only in *KRAS*-mutated cells.

**Figure 5:**
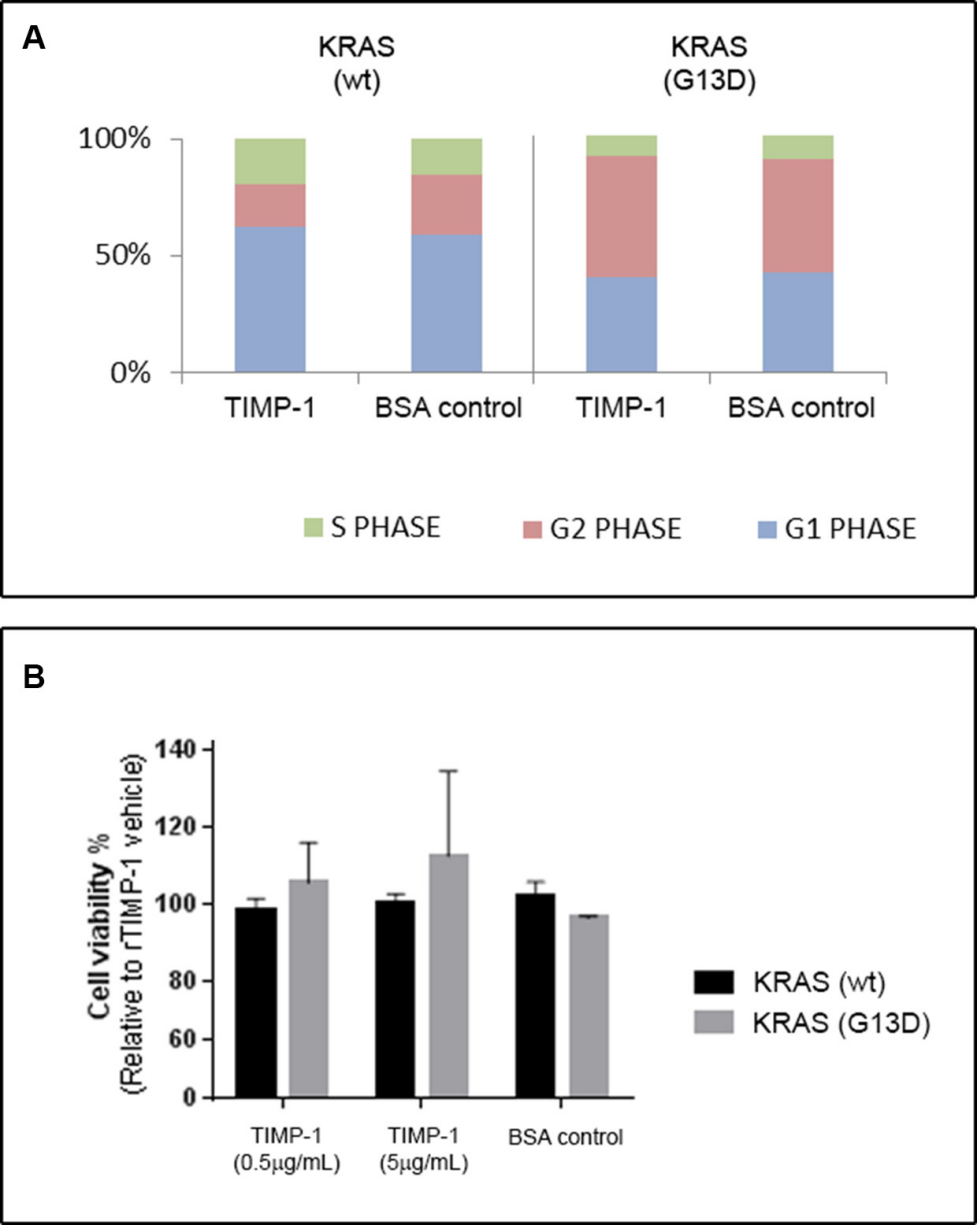
TIMP-1 does not affect cell cycle progression DLD-1 clones bearing a *KRAS*-mutated allele (KRAS G13D) or a wild-type allele (KRAS wt), were grown in the presence of either rTIMP-1 (5 mg/mL) or BSA (control) and subsequently either subjected to (**A**) cell cycle analysis or (**B**) cell proliferation. (A) The cell cycle phase distributions after exposure to TIMP-1 or BSA for 24 h were evaluated by FACS and are presented as histograms. (B) Cells treated with rTIMP-1 (0.5 or 5 μg/mL) for 48 h and cell proliferation was assessed by a crystal violet proliferation assay. Data represents the mean ± SEM of three independent assays.

Given that TIMP-1 can promote cell proliferation and survival, we examined the cell cycle profile of *KRAS* wt and *KRAS* G13D mutated CRC cells stimulated or not with TIMP-1. We found that, although there were significant differences in cell-cycle profile between the two cell lines, exposure to TIMP-1 did not alter the cell-cycle profile of either cell line to any noticeable degree (Figure 5A). We then determined cell viability by the crystal violet assay. As shown in Figure 5B, exposure to TIMP-1 did not change cell viability of *KRAS* wt, but we could observe a dose-dependent effect on cell viability of *KRAS* G13D mutated CRC cells. Although, this effect was not statistical significant, the trend towards increased cell viability of *KRAS* G13D mutated CRC cells, but not *KRAS* wt cells, was consistent with the effects we had observed on cell invasion and colony formation.

### Identification of KRAS-mutation specific targets upon cell exposure to TIMP-1

To address the molecular mechanisms underlying the *KRAS* mutation dependent, TIMP-1 associated effects on CRC cells, we performed gene expression profiling of *KRAS* wt and *KRAS* G13D mutated CRC cells stimulated or not with TIMP-1. We then performed a supervised comparative analysis of the results using a predefined set of criteria: to be considered, genes should show differential expression in *KRAS* G13D mutated cells upon stimulation with TIMP-1, but no significant differences in *KRAS* wt cells. In addition, their baseline expression in *KRAS* G13D mutated cells should not be significantly different from *KRAS* wt cells. Finally, genes should be categorized under the gene ontology term “Ras protein signal transduction”. We identified five genes (Table 3) that fulfilled these criteria: *RAF1* (RAF proto-oncogene serine/threonine-protein kinase), *MAP3K11* (*MLK3*; mitogen-activated protein kinase kinase kinase 11), *MAP3K3* (*MEKK3*; mitogen-activated protein kinase kinase kinase 3), *MAP3K6* (*ASK2*; mitogen-activated protein kinase kinase kinase 6), and *MAP4K2* (*GCK*; mitogen-activated protein kinase kinase kinase kinase 2). Suggestively, the five genes we identified in this manner are all involved in the RAS-JNK-p38 MAPK signaling axis (Figure S3), one of the signaling pathways related to RAS signaling and a key transduction pathway in CRC [41, 42], thus providing a molecular elucidation for our observations. A more comprehensive analysis of the transcriptomic, as well as functional proteomic changes, that take place in *KRAS* G13D mutated cells upon stimulation with TIMP-1 is ongoing, and will be reported elsewhere.

**Table 3:** Genes differentially up-regulated in *KRAS*-mutated cells in the presence of rTIMP-1 (5 μg/mL) as compared to *KRAS*-wild-type bearing cells

Gene	Log2FoldChange			*P*-value		
	KRAS wt vs KRAS G13D	KRAS wt vs KRAS wt + TIMP-1	KRAS G13D vs KRAS G13D + TIMP-1	KRAS wt vs KRAS G13D	KRAS wt vs KRAS wt + TIMP-1	KRAS G13D vs KRAS G13D + TIMP-1
*RAF1*	−0.05	0.01	1.93	0.9291	0.9834	0.0068
*MAP3K11*	−0.58	0.05	2.20	0.3576	0.9340	0.0036
*MAP3K3*	−0.21	−0.09	1.64	0.6709	0.8587	0.0056
*MAP3K6*	−0.40	0.03	2.10	0.5012	0.9612	0.0035
*MAP4K2*	0.18	0.22	1.63	0.7054	0.6521	0.0047

## DISCUSSION

Our laboratory has throughout the years published a number of articles that point to TIMP-1 as a potentially valuable biological marker, be it for early detection, prognosis, or predictive of therapy outcome in CRC [27, 29, 30, 43, 44]. TIMP-1 is an endogenous inhibitor of metalloproteinases, a family of proteins that play a role in tumor invasion and metastases. But in addition to its canonical MMP-inhibitory function, TIMP-1 can stimulate cell proliferation and prevent apoptosis, independently of the presence of the MMP inhibitory domain; it has been shown that binding of TIMP-1 to the cell surface can signal through RAS, to activate the PI3K/AKT signal pathways, and also that EGF signaling can induce TIMP-1 expression [36, 37, 45, 46]. The multiple level of interactions that exist in EGF signaling, RAS, and cell survival, proliferation and invasion, indicated that high plasma TIMP-1 levels may interact with EGFR signaling and thereby affect the anti-tumor effects of EGFR inhibitors. We investigated the association between plasma TIMP-1 levels and clinical outcome in patients treated with a regimen based on an oxaliplatin backbone adding (or not) cetuximab (the NORDIC VII Study). The results, presented here, showed an association between high plasma TIMP-1 levels and benefit from adding cetuximab to the standard (FOLFOX) treatment regimen in patients bearing *KRAS* mutated tumors (Figure 1 and Table 2), identifying plasma TIMP-1 levels as a novel predictive biomarker for cetuximab response in *KRAS* mutated tumors (Figure 2). We were also able to confirm a strong prognostic effect of plasma TIMP-1 levels irrespective of treatment (Figure 1 and Table 2), which reiterates previously reported data that plasma TIMP-1 is a prognostic biomarker in mCRC patients receiving oxaliplatin-containing treatment [30]. But it is not straightforward to reconcile these observations with TIMP-1′s biological functions. Neither of the biological activities previously ascribed to TIMP-1, be it the canonical MMP-dependent or the MMP-independent function, can directly explain the association we found between high plasma TIMP-1 levels and benefit from cetuximab treatment in *KRAS* mutated tumors. If anything, the PI3-K/AKT associated pro-survival effect should be deleterious for cetuximab-treated patients, as well as being independent of *KRAS* status. To address this issue, we examined the expression and cellular effect(s) of TIMP-1 in CRC cells upon stimulation with EGF, and in cells bearing, or not, a *KRAS*-mutated allele. We found that TIMP-1 expression could be stimulated by exposing CRC cells to EGF ligand in a dose-dependent manner (Figure 3). This effect was directly related to expression levels of EGFR (Figure S2), showing that the EGF-ligand/EGFR signaling axis plays an important regulatory role in TIMP-1 expression in CRC cells. We could also ascertain that TIMP-1 promoted colony formation and cell invasion in *KRAS*-mutated cells, but not in *KRAS* wt cells (Figure 4), consistent with potentiation by TIMP-1 of aggressive behavior specifically in *KRAS* mutated cells. This bimodal interaction between EGF-EGFR signaling and TIMP-1 expression on the one hand, and TIMP-1 mediated stimulation of cell invasion, specifically on *KRAS*-mutated cells, on the other hand, suggested that the predictive value of TIMP-1 we observed is the outcome of two additive effects; first that TIMP-1 expression is under control of EGFR-signaling, independently of the RAS/MAPK-axis (Figures 3 and S2), and secondly that TIMP-1 potentiates an aggressive behavior in *KRAS* mutated cells but not *KRAS* wt cells (Figure 4). The latter effect is presumably brought about by the increased expression of downstream effectors in the RAS- JNK- p38 MAPK pathway we observed in *KRAS* G13D mutated cells stimulated with TIMP-1 (Table 3). When tumor cells are exposed to cetuximab, expression of TIMP-1 will be inhibited, irrespective of *KRAS* status. This will not have a noticeable effect on *KRAS* wild-type tumor cells, but will reduce the stimulatory drive of extracellular TIMP-1 on *KRAS* mutated tumor cells. These two levels of interaction are required to fully account for the predictive value of TIMP-1 specifically in cetuximab-treated *KRAS*-mutated patients. From a mechanistic point of view, and taking into account the results of our gene expression analysis, the effect of TIMP-1 will not be particularly striking in patients treated with cetuximab as monotherapy, but will do so whenever a chemotherapeutic component is used, as the effect on the JNK/p28 MAPK pathway will be significant under these conditions, which is in line with previous studies from our laboratories [29, 47].

It should be noted that, because our analysis was conducted retrospectively, and the NORDIC VII trial was not specifically designed, and powered, to assess the clinical activity of cetuximab in biomarker-specific subgroups, these results need to be validated in an independent cohort of patients with mCRC. In the meantime, this has proven to be a very difficult task, as the most recent clinical trials on anti-EGFR therapies exclude *KRAS*-mutated patients, and the original ones, which included *KRAS*-mutated patients, no longer have available biological material suitable for analysis of TIMP-1. Overall, our data provides a strong rationale to study the value of TIMP-1 as a predictive biomarker for benefit from EGFR-inhibition therapy in patients bearing *KRAS* mutated tumors and we are currently planning such a clinical study.

## MATERIALS AND METHODS

### Cell lines and culturing conditions

The colorectal cancer cell lines SW620 and Colo205 were purchased from the American Tissue Culture Collection (Rockville, MD, USA), while the HCT-15 and HT-29 were obtained from the NCI/Development Therapeutics Program. The DLD-1 cell line and matched pair of isogenic DLD-1 cell clones (KRAS G13D and KRAS wt) [38] were a kind gift from Bert Vogelstein (Howard Hughes Medical Institute, The Johns Hopkins Medical Institution, USA). DLD-1 and derivative clones were cultured in McCoy's 5A medium supplemented with 10% fetal bovine serum (FBS); all other lines were cultured in RPMI-1640 medium supplemented with 10% FBS. All cells were maintained in cell culture flasks (TPP, Transadingen, Switzerland) at 37°C in a humidified, 5% CO_2_ atmosphere under sterile conditions. All cell culture media were from Life Technologies (CA, USA), and plasticware was from Nunc (Thermo Fischer Scientific, USA). Cell identity was verified by short tandem repeat (STR) loci analysis at IdentiCell (Aarhus, Denmark). Cells were regularly tested for mycoplasma infection (Minerva Biolabs, Germany).

For EGF stimulation assays, cells were plated overnight, washed twice in PBS to remove serum remnants, and subsequently serum-starved in serum-free growth media for 24 h, after which fresh serum-deprived medium containing human recombinant EGF (rEGF) (Sigma Aldrich, MO, USA) at 10 or 50 ng/mL was added and cells cultured for an additional 24 h or 48 h period. As controls, equivalent concentrations of rEGF solvent (acetic acid) or 10% FBS were added in each case, respectively. When relevant, recombinant TIMP-1 [40] was added at the stated concentrations.

### SDS-PAGE and Western blot analysis

Whole cell extracts were obtained by lysis of 70–80% confluent cells. Briefly, cells were washed with ice-cold PBS before on-plate lysis with 250–500 μL M-PER Mammalian Protein Extraction Reagent (Thermo Scientific, MA, USA) containing Pierce Protease and Phosphatase Inhibitor Mini Tablets (Thermo Scientific, MA, USA). The lysates were centrifuged at 14000 × g for 10 min to remove cell debris and total protein concentrations of the samples were measured using a BCA protein Assay Kit (Novagen, CA, USA) according to manufacturer's instructions. For analysis, equivalent amounts of total protein (20 μg per well) were subjected to SDS-PAGE separation under reducing conditions. Resolved proteins were blotted onto 0.2 μm nitrocellulose membranes (Bio-Rad, CA, USA), and blocked in 5% milk or BSA (antibody-specific) in Tris-Buffered Saline and Tween 20 (TBS-T, 0.05%) for 1 hour at room temperature. The membranes were incubated overnight with relevant primary antibodies diluted in 5% milk blocking solution (p150^Glued^, and VT-7), or 5% BSA [phospho-AKT(Ser374), and AKT] in TBS-T at 4°C. After washing thrice for 10 min in TBS-T, followed by detection of immune complexes with corresponding horseradish peroxidase-labeled species specific antibodies (Dako, Denmark), detection of immune complexes was done using the Amersham ECL-Select Western Blotting detection reagent (GE Healthcare Life Sciences, NJ, USA) or Clarity Western ECL Substrate (Bio-Rad, CA, USA) according to manufacturer's instruction and images were captured with a BioSpectrum Imaging system (Ultra-Violet Products, CA, USA). The anti-p150^Glued^ antibody was from BD Biosciences (NJ, USA), the anti-TIMP-1 antibody was an in-house antibody (VT-7) previously described [48], and the anti- phospho-AKT (Ser374) and AKT antibodies were from Cell Signaling Technologies (MA, USA). Band intensity was evaluated by densitometric measurements with Image J software (National Institutes of Health).

### *In vitro* invasion assay

*In vitro* invasive potential was determined using Corning^®^ Biocoat^™^ Matrigel^®^ invasion chambers (VWR, Radnor, PA, USA) according to manufacturer's instructions. Briefly, cells were serum-starved for 24 h, harvested by trypsin-EDTA treatment, resuspended in serum-free medium containing trypsin inhibitor according to manufacturer's instructions (Sigma Aldrich, MO, USA), and centrifuged to form a cell pellet (Sigma Aldrich, MO, USA). The cells were washed twice in serum-free medium, resuspended, and inoculated at a density of 500000 cells/500 μL/chamber onto an 8 μm pore Matrigel^®^-coated membrane. The inserts were inset in 24-well plates, with each well filled with 750 μl of medium containing 50 ng/mL EGF as chemoattractant, and subsequently incubated at 37°C for 14 h. After incubation, non-invasive cells were carefully scraped off with a cotton swab and invasive cells were fixed in 100% methanol, stained with 10% Giemsa, and counted with a light microscope by two independent observers. Four independent experiments were performed, each with technical duplicates, in all experimental conditions. Mean values of the duplicates were used for statistical analysis.

### Soft agar clonogenic assay

To assess a potential differential biological effect of TIMP-1 on anchorage-independent growth in *KRAS* mutated cells, a double layer soft agar assay was performed. Monolayer cultures of DLD-1 isogenic cells (*KRAS* (wt) and (*KRAS* (G13D)) were prepared into single-cell suspensions using 0.01% trypsin-EDTA. The cells were suspended in growth medium containing 0.18% low melting temperature agar (Sigma Aldrich, MO, USA) supplemented with either 5 μg/mL BSA or rTIMP-1, seeded at a density of 800 cells/750 μL/well on top of a solidified bottom layer of 0.75% agar in growth medium with 5.0 μg/mL rTIMP-1 or BSA (control) in 12-well plates. The following day 500 μL growth medium containing TIMP-1 or BSA in corresponding concentrations was added. Each condition was set up in triplicate and three independent assays were performed. Visible colonies were counted independently by two observers after 21 and 28 days.

### Cell viability and FACS assays

DLD-1 isogenic cells KRAS (wt/−) and KRAS (−/G13D) were plated on 6-well tissue culture plates, washed twice in PBS to remove serum, and serum starved in 2 mL McCoy's 5A medium for additional 24 h to synchronize the cells. rTIMP-1 (5 μg/mL) or BSA were added and cells incubated for 24 h. Cells were seeded at concentrations such that they would reach approximately 70% confluency when measured, corresponding to 200000 and 220000 cells/well for KRAS (wt) and KRAS (G13D), respectively. Cells were collected by trypsinisation, washed briefly in serum-free McCoy's 5A medium containing trypsin inhibitor (diluted 1:100), and fixed and permeabilized by adding 700 μL ice-cold 96% ethanol on ice with intermittent vortexing every 10 min. Prior to analysis, cell samples were centrifuged at 1200 rpm for 5 min and cell pellets resuspended in 200 μL propidium iodide (PI) containing buffer with RNase, and incubated for 30 min. The samples were analysed in a flow cytometer (BD FACSVerse) set for PI acquisition. We used pulse-width/pulse-area to discriminate between cells in G2/M and cell doublets with the intent to gate out the latter. The data was analysed using FlowJo software.

For cell viability/proliferation we used a standard crystal violet assay. Briefly, cells were plated at a density of 25000 cells/well in 24-well plates, and grown in serum-free medium for 4 h. TIMP-1 was then added to the medium to reach final concentrations of 0.5 μg/mL or 5 μg/mL. Two controls were run in parallel: 5 μg/mL BSA (Roche Diagnostics, Basel, Switzerland) and PBS (rTIMP-1 vehicle). Cells were allowed to proliferate for 48h, washed thrice with PBS, and stained with 200 μL of a 0.5% solution of crystal violet.

### Gene expression analysis

DLD-1 isogenic cells (*KRAS* (wt/−) and (*KRAS* (−/G13D)) were plated and serum-starved for 3 h in medium containing 1% FBS, prior to exposure to TIMP-1. Cells were stimulated with 5 μg/mL rTIMP-1 for 4 h, washed and RNA was extracted using RNeasy mini-kit according to the manufacturer's protocol (Qiagen, CA, USA).

Samples were prepared for analysis using Low Input Quick Amp Labeling Kit, one-color (Agilent Technologies, CA, USA) according to manufacturer's instructions. Labelled cDNA was purified and hybridized to the Agilent one-color human whole genome microarray chip, which was analyzed using Agilent Feature Extraction Software (Agilent Technologies). Samples were clustered by calculating pairwise distances followed by clustering by the ‘ward’ method. Log2FoldChange of KRAS wt vs KRAS G13D mutated cells, as well as of untreated vs treated cells, and their corresponding *p*-values were calculated using moderated *t*-test. Genes were considered significantly differentially expressed if *p*-value < 0.0075 and Log2FoldChange > +/−1.

### Patient characteristics and analysis

In the NORDIC VII study [34] 566 patients with mCRC were included from 32 Nordic centers. The baseline demographic characteristics of the 426 patients with a pre-treatment plasma TIMP-1 measurement are shown in Table 1. All patients provided written informed consent, and the study (including biomarker analyses) was approved by the Regional Ethics Committee (VEK ref. 20050053).

Patients were randomized between; Nordic FLOX: 5-FU i.v. bolus 500 mg/m^2^ and folinic acid 60 mg/m^2^ day 1–2, oxaliplatin 85 mg/m^2^ day 1 every two week until progression (arm A); Nordic FLOX plus cetuximab (400 mg/m^2^ day 1, then 250 mg/m^2^ weekly) until progression (arm B) or Nordic FLOX + cetuximab for 16 weeks, and weekly cetuximab as maintenance treatment until progression (arm C). Main inclusion criteria were: histologically confirmed mCRC (adenocarcinomas); age > 18 years and < 75 years; WHO performance status (PS) ≤ 2; no prior chemotherapy for mCRC, non-resectable and measurable disease according to the Response Evaluation Criteria in Solid Tumors (RECIST version 1.0); last adjuvant chemotherapy ≥ 6 months before inclusion; no previous oxaliplatin treatment; adequate haematological, renal and liver function.

The patients were treated until disease progression and followed until death or 30th April 2009. Pre-treatment plasma sample were available from 426 (75%) patients at baseline. Further details about the study have been published [34]. Survival probabilities for overall survival were estimated by the Kaplan-Meier method and tests for differences between strata were done using the log-rank statistic. *P*-values less than 5% were considered statistically significant. Statistical calculations were performed using SAS (version 9.2, SAS Institute, Cary, NC, USA) and R [49].

### Statistical analysis of NORDIC VII data

The primary clinical endpoint for this biomarker study was overall survival (OS) determined as the time from randomization to treatment in NORDIC VII study to time of death by any causes. The median follow-up time was 37 month (24–53 months). Cases in which patients were alive at this date were censored. Secondary endpoint was progression-free survival (PFS) (primary endpoint of the NORDIC VII study) defined as the time from randomization until objective disease progression. Descriptive statistics are presented as median levels and ranges. Analyses of measurements for PFS and OS were done using the Cox proportional hazards model. As the analyses performed comparing treatment arms did not reveal any substantial differences in terms of OS and PFS between the original study population and this subset of patients (please see below), we found it justified to pool arms B and C (i.e. + cetuximab treatment) for the statistical analyses. Thus, patients were stratified as receiving cetuximab or not, i.e. arm B and C versus arm A.

Survival probabilities for OS were estimated by the Kaplan-Meier method and tests for differences between strata were done using the log-rank statistic. Graphical presentation using Kaplan-Meier estimates of PFS and OS was shown grouping patients in tertiary TIMP-1 levels. Multivariable analysis of PFS and OS was done using the Cox proportional hazard model. TIMP-1 concentrations in plasma were entered by the actual value on the log scale (base 2). Missing values for CEA (*n* = 27), CRP (*n* = 22), *BRAF* (*n* = 101) and *KRAS* (*n* = 62) were categorized separately and included in the final multivariable analysis. The final model included a three way interaction term (treatment +/− cetuximab +/− x *KRAS* mutational status x plasma TIMP-1). The model was assessed using Schoenfeld and Martingale residuals. In particular, the linearity assumption for plasma TIMP-1 on the log scale was evaluated using the supremum test for the cumulated martingales [50]. The results yielded *P* > 0.05 testing for the linearity of plasma TIMP-1 on the log scale. Ten-fold cross validation performed in order to assess over-fitting [51] showed almost similar results for the training and test sets (data not shown). *P*-values less than 5% were considered statistically significant. Statistical calculations were performed using SAS (version 9.2, SAS Institute, Cary, NC, USA) and R [49]. The results of this project are reported in accordance with the REMARK guidelines [52].

### Biomarker analyses

Total plasma TIMP-1 levels (free and in complex with matrix metalloproteinase) were determined using the MAC15 antibody kinetic enzyme-linked immunosorbent assay (ELISA) as described [35]. Duplicate measurements were carried out and the mean values were used for statistical analysis. The mean intra-assay coefficient of variation (CV) was 5.1% (range 1.5%–9.8%) and the inter-assay CV was 6.7%. Data on serum carcinoembryonic antigen (CEA), C-reactive protein (CRP), *KRAS* and *BRAF* mutational status of the tumor, WHO performance status (PS), and number of metastatic sites were retrieved from the original study report [34].

### Statistical analysis of *in vitro* data

One-way ANOVA tests were conducted to compare the mean intensity of the bands of western blots within one cell line after treating the cells with rEGF. For colony formation and Boyden chamber assyas, a two-way analysis of variance (ANOVA) was used to compare the mean of samples that are influenced by two variables: namely i) cell line (KRAS status) and ii) the experimental condition (rTIMP-1 or BSA). Multiple comparisons post tests were applied to counteract the risk of type I errors when conducting multiple comparisons. GraphPad Prism (GraphPad Software, USA) was used for one-way and two-way analyses of variance (ANOVA) and P-values were adjusted for multiplicity.

## SUPPLEMENTARY MATERIALS FIGURES



## References

[1] Jorissen RN, Walker F, Pouliot N, Garrett TP, Ward CW, Burgess AW (2003). Epidermal growth factor receptor: mechanisms of activation and signalling. Experimental cell research.

[2] Holbro T, Civenni G, Hynes NE (2003). The ErbB receptors and their role in cancer progression. Experimental cell research.

[3] Hynes NE, MacDonald G (2009). ErbB receptors and signaling pathways in cancer. Current opinion in cell biology.

[4] Normanno N, De Luca A, Bianco C, Strizzi L, Mancino M, Maiello MR, Carotenuto A, De Feo G, Caponigro F, Salomon DS (2006). Epidermal growth factor receptor (EGFR) signaling in cancer. Gene.

[5] Klein S, Levitzki A (2009). Targeting the EGFR and the PKB pathway in cancer. Current opinion in cell biology.

[6] Ciardiello F, Tortora G (2008). EGFR antagonists in cancer treatment. The New England journal of medicine.

[7] Tol J, Punt CJ (2010). Monoclonal antibodies in the treatment of metastatic colorectal cancer: a review. Clinical therapeutics.

[8] Bleeker WK, Lammerts van Bueren JJ, van Ojik HH, Gerritsen AF, Pluyter M, Houtkamp M, Halk E, Goldstein J, Schuurman J, van Dijk MA, van de Winkel JG, Parren PW (2004). Dual mode of action of a human anti-epidermal growth factor receptor monoclonal antibody for cancer therapy. Journal of immunology.

[9] Monteverde M, Milano G, Strola G, Maffi M, Lattanzio L, Vivenza D, Tonissi F, Merlano M, Lo Nigro C (2015). The relevance of ADCC for EGFR targeting: A review of the literature and a clinically-applicable method of assessment in patients. Crit Rev Oncol Hematol.

[10] Nakadate Y, Kodera Y, Kitamura Y, Shirasawa S, Tachibana T, Tamura T, Koizumi F (2014). KRAS mutation confers resistance to antibody-dependent cellular cytotoxicity of cetuximab against human colorectal cancer cells. International journal of cancer.

[11] Lievre A, Bachet JB, Le Corre D, Boige V, Landi B, Emile JF, Cote JF, Tomasic G, Penna C, Ducreux M, Rougier P, Penault-Llorca F, Laurent-Puig P (2006). KRAS mutation status is predictive of response to cetuximab therapy in colorectal cancer. Cancer research.

[12] De Roock W, Claes B, Bernasconi D, De Schutter J, Biesmans B, Fountzilas G, Kalogeras KT, Kotoula V, Papamichael D, Laurent-Puig P, Penault-Llorca F, Rougier P, Vincenzi B (2010). Effects of KRAS, BRAF, NRAS, and PIK3CA mutations on the efficacy of cetuximab plus chemotherapy in chemotherapy-refractory metastatic colorectal cancer: a retrospective consortium analysis. The lancet oncology.

[13] De Roock W, Jonker DJ, Di Nicolantonio F, Sartore-Bianchi A, Tu D, Siena S, Lamba S, Arena S, Frattini M, Piessevaux H, Van Cutsem E, O’Callaghan CJ, Khambata-Ford S (2010). Association of KRAS p. G13D mutation with outcome in patients with chemotherapy-refractory metastatic colorectal cancer treated with cetuximab. JAMA.

[14] Van Cutsem E, Kohne CH, Hitre E, Zaluski J, Chang Chien CR, Makhson A, D’Haens G, Pinter T, Lim R, Bodoky G, Roh JK, Folprecht G, Ruff P (2009). Cetuximab and chemotherapy as initial treatment for metastatic colorectal cancer. The New England journal of medicine.

[15] Douillard JY, Oliner KS, Siena S, Tabernero J, Burkes R, Barugel M, Humblet Y, Bodoky G, Cunningham D, Jassem J, Rivera F, Kocakova I, Ruff P (2013). Panitumumab-FOLFOX4 treatment and RAS mutations in colorectal cancer. The New England journal of medicine.

[16] Peeters M, Douillard JY, Van Cutsem E, Siena S, Zhang K, Williams R, Wiezorek J (2013). Mutant KRAS codon 12 and 13 alleles in patients with metastatic colorectal cancer: assessment as prognostic and predictive biomarkers of response to panitumumab. Journal of clinical oncology.

[17] Mao C, Huang YF, Yang ZY, Zheng DY, Chen JZ, Tang JL (2013). KRAS p. G13D mutation and codon 12 mutations are not created equal in predicting clinical outcomes of cetuximab in metastatic colorectal cancer: a systematic review and meta-analysis. Cancer.

[18] Kumar SS, Price TJ, Mohyieldin O, Borg M, Townsend A, Hardingham JE (2014). KRAS G13D Mutation and Sensitivity to Cetuximab or Panitumumab in a Colorectal Cancer Cell Line Model. Gastrointestinal cancer research.

[19] Yoon HH, Tougeron D, Shi Q, Alberts SR, Mahoney MR, Nelson GD, Nair SG, Thibodeau SN, Goldberg RM, Sargent DJ, Sinicrope FA, Alliance for Clinical Trials in O (2014). KRAS codon 12 and 13 mutations in relation to disease-free survival in BRAF-wild-type stage III colon cancers from an adjuvant chemotherapy trial (N0147 alliance). Clinical cancer research.

[20] Sorensen NM, Schrohl AS, Jensen V, Christensen IJ, Nielsen HJ, Brunner N (2008). Comparative studies of tissue inhibitor of metalloproteinases-1 in plasma, serum and tumour tissue extracts from patients with primary colorectal cancer. Scandinavian journal of gastroenterology.

[21] Wurtz SO, Schrohl AS, Mouridsen H, Brunner N (2008). TIMP-1 as a tumor marker in breast cancer—an update. Acta oncologica.

[22] Jiang Y, Goldberg ID, Shi YE (2002). Complex roles of tissue inhibitors of metalloproteinases in cancer. Oncogene.

[23] Chirco R, Liu XW, Jung KK, Kim HR (2006). Novel functions of TIMPs in cell signaling. Cancer metastasis reviews.

[24] Moller Sorensen N, Vejgaard Sorensen I, Ornbjerg Wurtz S, Schrohl AS, Dowell B, Davis G, Jarle Christensen I, Nielsen HJ, Brunner N (2008). Biology and potential clinical implications of tissue inhibitor of metalloproteinases-1 in colorectal cancer treatment. Scandinavian journal of gastroenterology.

[25] Waas ET, Hendriks T, Lomme RM, Wobbes T (2005). Plasma levels of matrix metalloproteinase-2 and tissue inhibitor of metalloproteinase-1 correlate with disease stage and survival in colorectal cancer patients. Diseases of the colon and rectum.

[26] Curran S, Dundas SR, Buxton J, Leeman MF, Ramsay R, Murray GI (2004). Matrix metalloproteinase/tissue inhibitors of matrix metalloproteinase phenotype identifies poor prognosis colorectal cancers. Clinical cancer research.

[27] Holten-Andersen MN, Stephens RW, Nielsen HJ, Murphy G, Christensen IJ, Stetler-Stevenson W, Brunner N (2000). High preoperative plasma tissue inhibitor of metalloproteinase-1 levels are associated with short survival of patients with colorectal cancer. Clinical cancer research.

[28] Yukawa N, Yoshikawa T, Akaike M, Sugimasa Y, Takemiya S, Yanoma S, Imada T, Noguchi Y (2004). Prognostic impact of tissue inhibitor of matrix metalloproteinase-1 in plasma of patients with colorectal cancer. Anticancer research.

[29] Sorensen NM, Bystrom P, Christensen IJ, Berglund A, Nielsen HJ, Brunner N, Glimelius B (2007). TIMP-1 is significantly associated with objective response and survival in metastatic colorectal cancer patients receiving combination of irinotecan, 5-fluorouracil, and folinic acid. Clinical cancer research.

[30] Frederiksen C, Qvortrup C, Christensen IJ, Glimelius B, Berglund A, Jensen BV, Nielsen SE, Keldsen N, Nielsen HJ, Brunner N, Pfeiffer P (2011). Plasma TIMP-1 levels and treatment outcome in patients treated with XELOX for metastatic colorectal cancer. Annals of oncology.

[31] Hilska M, Roberts PJ, Collan YU, Laine VJ, Kossi J, Hirsimaki P, Rahkonen O, Laato M (2007). Prognostic significance of matrix metalloproteinases-1, -2, -7 and -13 and tissue inhibitors of metalloproteinases-1, -2, -3 and -4 in colorectal cancer. International journal of cancer.

[32] Fu ZY, Lv JH, Ma CY, Yang DP, Wang T (2011). Tissue inhibitor of metalloproteinase-1 decreased chemosensitivity of MDA-435 breast cancer cells to chemotherapeutic drugs through the PI3K/AKT/NF-small ka, CyrillicB pathway. Biomedicine & pharmacotherapy.

[33] Toricelli M, Melo FH, Peres GB, Silva DC, Jasiulionis MG (2013). Timp1 interacts with beta-1 integrin and CD63 along melanoma genesis and confers anoikis resistance by activating PI3-K signaling pathway independently of Akt phosphorylation. Molecular cancer.

[34] Tveit KM, Guren T, Glimelius B, Pfeiffer P, Sorbye H, Pyrhonen S, Sigurdsson F, Kure E, Ikdahl T, Skovlund E, Fokstuen T, Hansen F, Hofsli E (2012). Phase III trial of cetuximab with continuous or intermittent fluorouracil, leucovorin, and oxaliplatin (Nordic FLOX) versus FLOX alone in first-line treatment of metastatic colorectal cancer: the NORDIC-VII study. Journal of clinical oncology.

[35] Holten-Andersen MN, Murphy G, Nielsen HJ, Pedersen AN, Christensen IJ, Hoyer-Hansen G, Brunner N, Stephens RW (1999). Quantitation of TIMP-1 in plasma of healthy blood donors and patients with advanced cancer. British journal of cancer.

[36] Qiu Q, Yang M, Tsang BK, Gruslin A (2004). EGF-induced trophoblast secretion of MMP-9 and TIMP-1 involves activation of both PI3K and MAPK signalling pathways. Reproduction.

[37] Soula-Rothhut M, Coissard C, Sartelet H, Boudot C, Bellon G, Martiny L, Rothhut B (2005). The tumor suppressor PTEN inhibits EGF-induced TSP-1 and TIMP-1 expression in FTC-133 thyroid carcinoma cells. Experimental cell research.

[38] Yun J, Rago C, Cheong I, Pagliarini R, Angenendt P, Rajagopalan H, Schmidt K, Willson JK, Markowitz S, Zhou S, Diaz LA, Velculescu VE, Lengauer C (2009). Glucose deprivation contributes to the development of KRAS pathway mutations in tumor cells. Science.

[39] Botta GP, Reichert M, Reginato MJ, Heeg S, Rustgi AK, Lelkes PI (2013). ERK2-regulated TIMP1 induces hyperproliferation of K-Ras(G12D)-transformed pancreatic ductal cells. Neoplasia.

[40] Vinther L, Lademann U, Andersen EV, Hojrup P, Thaysen-Andersen M, Krogh BO, Viuff B, Brunner N, Stenvang J, Moreira JM (2014). Purification and characterization of bioactive his6-tagged recombinant human tissue inhibitor of metalloproteinases-1 (TIMP-1) protein expressed at high yields in mammalian cells. Protein expression and purification.

[41] Fang JY, Richardson BC (2005). The MAPK signalling pathways and colorectal cancer. Lancet Oncol.

[42] Grossi V, Peserico A, Tezil T, Simone C (2014). p38alpha MAPK pathway: a key factor in colorectal cancer therapy and chemoresistance. World J Gastroenterol.

[43] Holten-Andersen M, Christensen IJ, Nilbert M, Bendahl PO, Nielsen HJ, Brunner N, Fernebro E, Receptor E, Biomarker G (2004). Association between preoperative plasma levels of tissue inhibitor of metalloproteinases 1 and rectal cancer patient survival. a validation study. European journal of cancer.

[44] Nielsen HJ, Brunner N, Jorgensen LN, Olsen J, Rahr HB, Thygesen K, Hoyer U, Laurberg S, Stieber P, Blankenstein MA, Davis G, Dowell BL, Christensen IJ (2011). Plasma TIMP-1 and CEA in detection of primary colorectal cancer: a prospective, population based study of 4509 high-risk individuals. Scandinavian journal of gastroenterology.

[45] Wang T, Yamashita K, Iwata K, Hayakawa T (2002). Both tissue inhibitors of metalloproteinases-1 (TIMP-1) and TIMP-2 activate Ras but through different pathways. Biochem Biophys Res Commun.

[46] Akahane T, Akahane M, Shah A, Thorgeirsson UP (2004). TIMP-1 stimulates proliferation of human aortic smooth muscle cells and Ras effector pathways. Biochem Biophys Res Commun.

[47] Bystrom P, Berglund A, Nygren P, Wernroth L, Johansson B, Larsson A, Glimelius B (2012). Evaluation of predictive markers for patients with advanced colorectal cancer. Acta oncologica.

[48] Moller Sorensen N, Dowell BL, Stewart KD, Jensen V, Larsen L, Lademann U, Murphy G, Nielsen HJ, Brunner N, Davis GJ (2005). Establishment and characterization of 7 new monoclonal antibodies to tissue inhibitor of metalloproteinases-1. Tumour biology.

[49] Team RC R: A language and environment for statistical computing.

[50] Lin DY, Wwi L. J., Ying Z (2013). Checking the Cox model with cumulative sums of martingale-based residuals. Biometrika.

[51] Harrell FE, Lee KL, Mark DB (1996). Multivariable prognostic models: issues in developing models, evaluating assumptions and adequacy, and measuring and reducing errors. Statistics in medicine.

[52] Altman DG, McShane LM, Sauerbrei W, Taube SE (2012). Reporting Recommendations for Tumor Marker Prognostic Studies (REMARK): explanation and elaboration. PLoS medicine.

