# Supplementary material for: TIMP-1 is under regulation of the EGF signaling axis and promotes an aggressive phenotype in KRAS-mutated colorectal cancer cells: A potential novel approach to the treatment of metastatic colorectal cancer
Source: Oncotarget. 2016 Aug 8;7(37):59441–57. doi: 10.18632/oncotarget.11118 (PMC5312323; doi:10.18632/oncotarget.11118)
Supplement: Supplementary file 1 [file oncotarget-07-59441-s001.pdf]

TIMP-1 is under regulation of the EGF signaling axis and promotes an aggressive phenotype in KRAS-mutated colorectal cancer cells: A potential novel approach to the treatment of metastatic colorectal cancer

Supplementary Materials

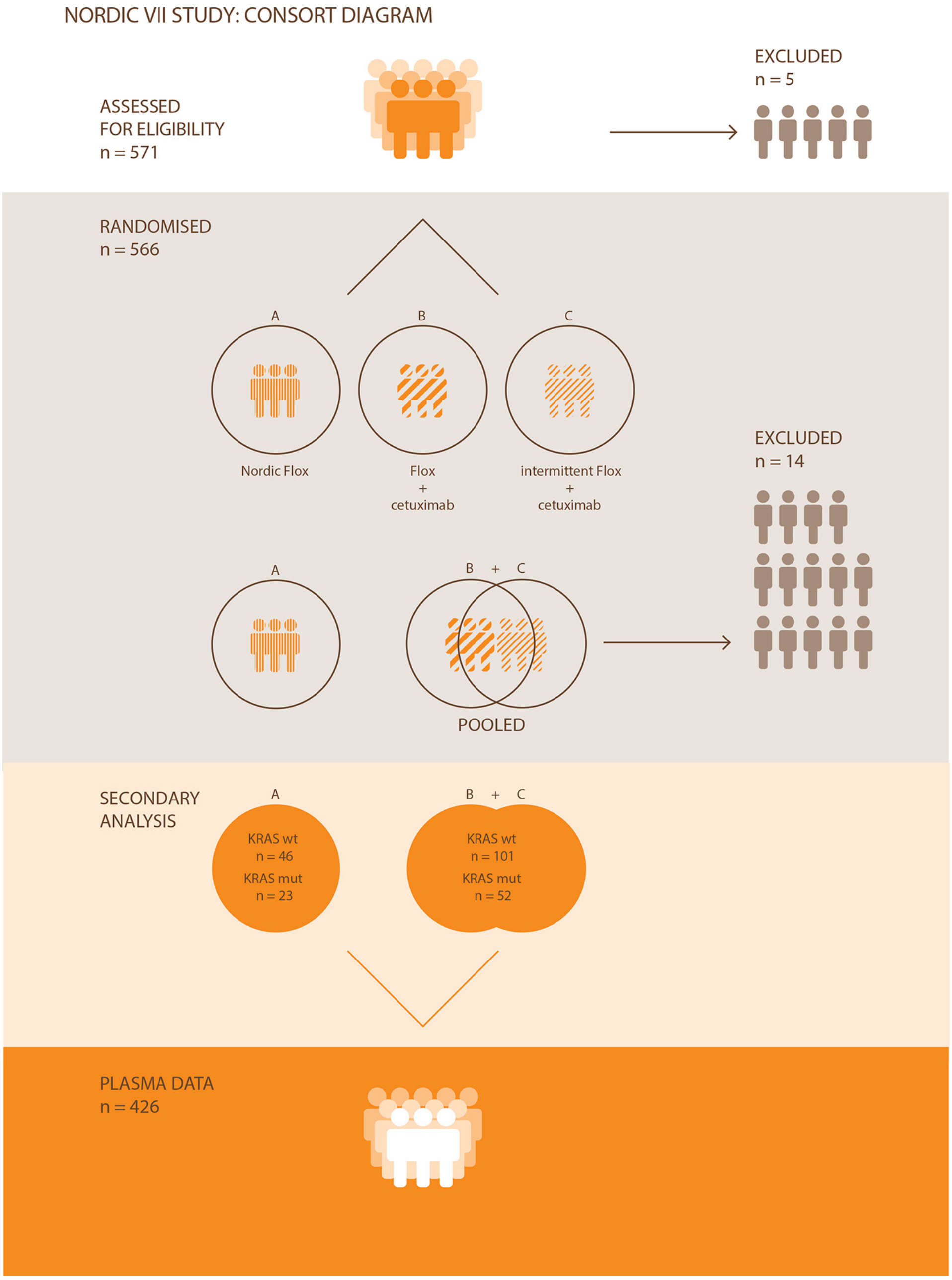

Supplementary Figure S1: Consort diagram indicating sample sizes at each stage during the study.

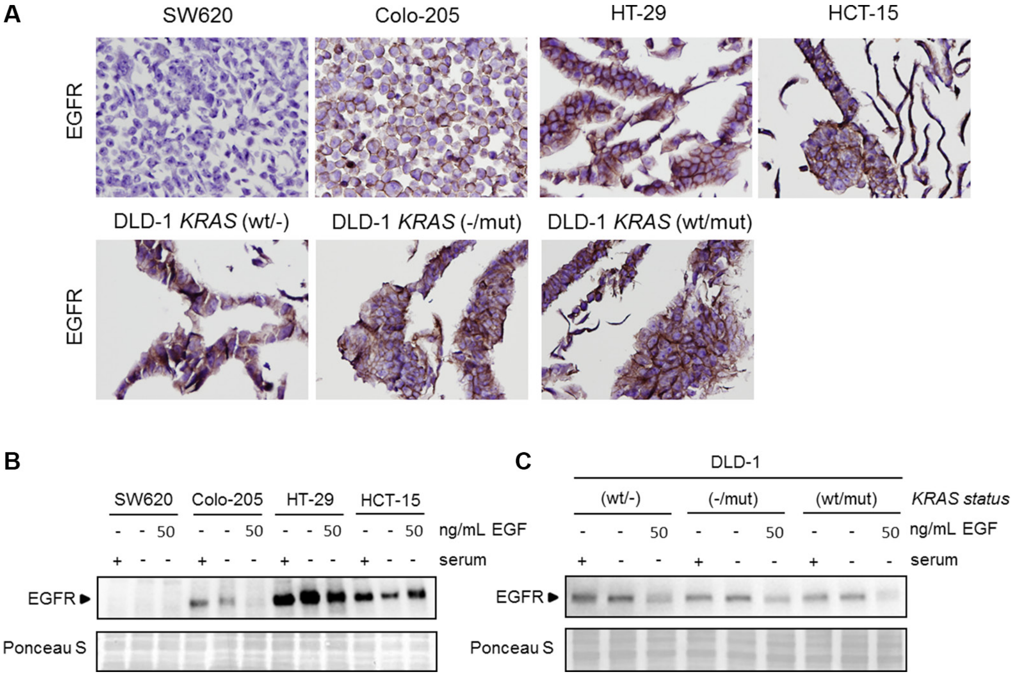

Supplementary Figure S2: EGFR expression in CRC cell lines. (**A**) Representative immunocytochemical images of EGFR expression in the cell lines under normal growth conditions. Images were captured at 40× magnification. (**B** and **C**) CRC cell lines were serum starved for 24 h and treated for 24 h with 50 ng/mL EGF. Controls with or without serum were cultured in parallel. Whole cell lysates were obtained, fractionated by SDS-PAGE, and immunoblotted for EGFR, Ponceau S staining was used as a loading control. Although DLD-1 cell lines were run on a different blot, the bands were developed simultaneously with the same exposure time. (B) Western blots of SW620, Colo-205, HT-29, and HCT-15 CRC cell lines; (C) Western blots of DLD KRAS isogenic cell lines and DLD-1 parental. Data presented are representative images.

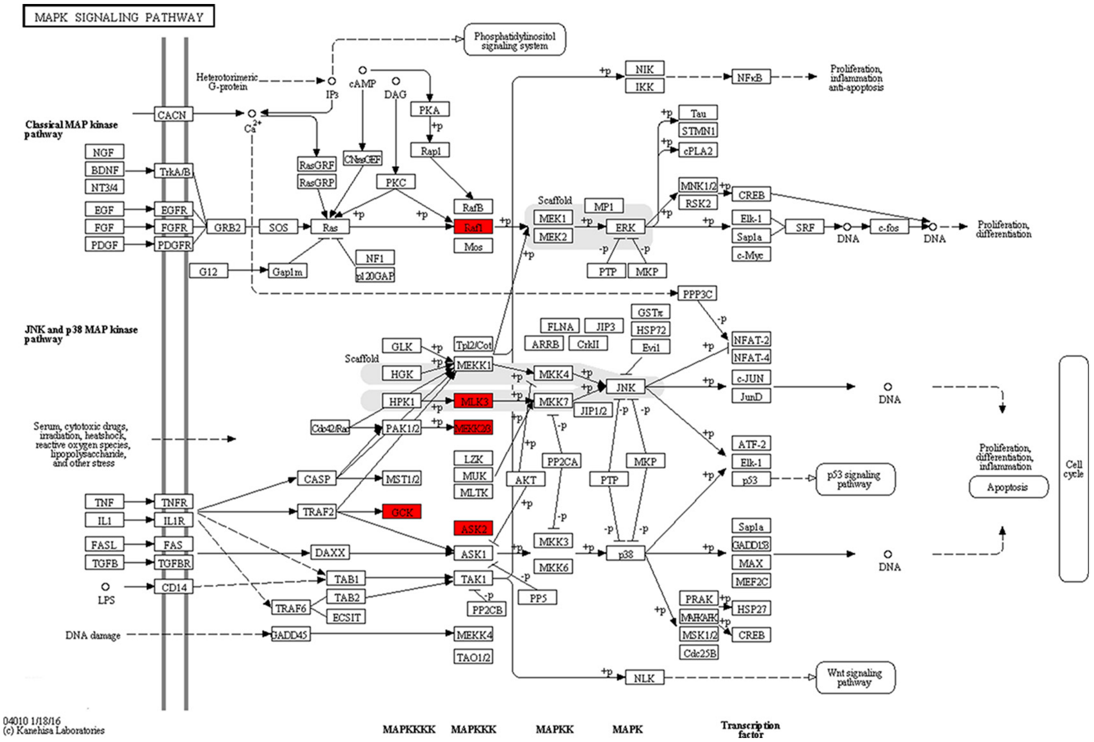

Supplementary Figure S3: Pathway analysis of genes deregulated in *KRAS* mutated cells in the presence of TIMP-1. Gene expression profiling of KRAS wt and KRAS G13D mutated CRC cells stimulated or not with TIMP-1 identified five genes as specifically deregulated in KRAS mutated cells in the presence of TIMP-1. These genes: *RAF1, MLK3, MEKK3, ASK2, and GCK* (highlighted in red) are shown in a section of the MAPKs KEGG pathway map (http://www.genome.jp/kegg/; accessed 05.02.2015).
